# Intergenerational Relations in the Climate Movement: Bridging the Gap toward a Common Goal

**DOI:** 10.3390/ijerph20010233

**Published:** 2022-12-23

**Authors:** Senjooti Roy, Liat Ayalon

**Affiliations:** Louis and Gabi Weisfeld School of Social Work, Bar-Ilan University, Ramat Gan 5290002, Israel

**Keywords:** intergenerational relations, climate change, intergenerational solidarity, intergenerational cooperation, intergenerational tension, intergenerational conflict

## Abstract

The call for climate action has attracted global support, especially among youth. Over time, climate discourse has turned increasingly hostile toward both younger and older generations, potentially engendering intergenerational conflict when intergenerational cooperation is most needed. Using a purposive sampling method, we selected a sample of 50 international influential individuals to gain an overview of the common views in current climate discourse to examine how they may impact intergenerational relations. We used a summative content analysis approach to analyze the different worldviews. The results are broadly divided into two categories—messages of intergenerational tension and messages of intergenerational solidarity. We found that although both types of messaging communicate similar facts and concerns, their presentation may either unite or divide different generations. Therefore, to work toward a common future for the benefit for all ages, it is essential for polarizing and accusatory sentiments to be replaced with messages of inclusivity and cooperation. This may be facilitated through intergenerational contact and interventions.

## 1. Introduction

Intergenerational relations are defined as reciprocal relationships between members of several generations comprising aging parents, adult children, grandchildren, and, sometimes, great-grandchildren. These relationships shape the cultural, social, economic, and educational experiences of the different generations. Intergenerational relations, especially long-term exchanges, are credited with wide-ranging benefits for both young and old, ranging from reducing loneliness, providing a sense of purpose, and enhancing quality of life among older adults to increasing feelings of empathy and reducing negative stereotypes about older adults among younger generations [1,2]. Sometimes, however, large-scale developments, such as the technological revolution, may increase disparities between generations and alter the norms that govern intergenerational relationships [3].

Intergenerational relations are guided by factors such as justice and equity [4,5]. They also consider the contexts of space and time within which these relations unfold. Moreover, demographic changes, both present trends and future projections, also affect such relationships. Positive intergenerational relations of solidarity and cooperation are grounded in respect for the challenges and opportunities associated with different stages of life and genuine concern toward the well-being of each age group. Negative intergenerational relations of tension and conflict pit different age groups against one another in competition for resources and recognition of socio-economic contributions [6].

In this article, we examine intergenerational relations in climate change discourse. A recent scoping review has highlighted the importance of intergenerational relations in the context of climate change [7]. However, the review concluded that much of the literature in the field is theoretical in nature. Most of the studies included in the review focused on solidarity between the generations. Intergenerational tension or conflict was reported in only two studies, but intergenerational differences in opinions and perceptions regarding climate realities were explored in eight studies. As such, we used the perspective of intergenerational relations proposed by Ayalon et al., 2022 [7] to examine contemporary discourse concerning climate change activists. This information is essential in order to develop a common ground between the generations and ensure solidarity in the climate change movement.

### 1.1. Climate Change and Intergenerational Exchange 

Although much of the climate discussion emphasizes the consideration of wealth, developmental status, and ethnic/racial identity to understand the effects of climate change on different populations, age and generation must also be recognized as significant sources of power differential [8]. Clearly, greenhouse gases emitted in the present by current generations will impact future generations. Children are often thought to be the most affected by climate change due to increased vulnerability to injury, disease, and extreme weather conditions [9]. Moreover, they are expected to suffer multiple consequences of climate change, such as natural disasters and shortages of food and water, for a substantially longer period, with these effects intensifying over time [10]. Additionally, it is expected that an entire generation’s gains in life expectancy may be compromised by climate change [11]. In the face of such dire prospects, members of the younger generation are well within their rights to demand stringent climate action from those in positions of power. However, when they protest against governments and international bodies, they are dismissed and belittled as being “misguided”, “idealistic”, or “mentally ill” [12]. 

A major source of intergenerational tension with regard to climate change stems from the strong sentiment that (older) adults are “greedy” and “selfish”, that they place their own interests above all else, and destroy the planet to accumulate vast amounts of wealth and power [13]. Despite such views, however, a comprehensive review has found limited differences between young and old people’s attitudes and knowledge concerning climate change [14]. A recent study undertaken in the United Kingdom has found almost no difference between the young and the old regarding their views about climate action. In fact, older people were more likely to believe that environmentally conscious actions have the potential to make a positive difference whereas younger people were more likely to be fatalistic [15]. Research has also shown that it is, in fact, older people who have been most affected by climate change because they are more susceptible to extreme heat waves, severe weather disruptions, pollution, and natural disasters [16,17,18]. In low- and middle-income countries, older adults are more likely to lose their livelihoods and food security, in addition to experiencing displacement and migration. Older adults around the world are also more vulnerable to resource poverty due to decreased income at older ages and may, therefore, be unable to protect themselves against climate change impacts [19]. Notably, to address climate change today, it is the current older generations that are expected to make sacrifices for a future of which they will not be a part [20]. Nevertheless, most older people aspire to leave a valuable legacy for future generations [17].

### 1.2. Intergenerational Relations, Ageism, and Health

Intergenerational relations, be they of conflict or solidarity, can greatly impact the physical, mental, and social health of individuals. Relationships of intergenerational conflict have the potential to engender ageism, both self-directed and from external sources. Ageism toward and among older people is associated with adverse health outcomes such as decreased quality of life, early death, slower recovery from disability, depression, social isolation, loneliness, and overall poorer physical and mental health [21]. A recent systematic review of the effects of ageism on older people’s health comprising over 7 million participants spanning 45 countries found that ageism led to significantly worse health outcomes across 11 domains in 95.5% of the studies [22]. Another study on the economic costs of ageism with respect to healthcare spending in the United States found that eight conditions alone accounted for over USD 63 billion in healthcare costs per year, with 17.04 million cases attributed to ageism [23].

While relationships of intergenerational conflict may give rise to ageism and negative health outcomes, relationships of intergenerational solidarity can foster cooperation and compassion, leading to positive health outcomes. For example, a study on intergenerational solidarity with over 28,000 participants comprising indigenous elders and indigenous people living off-reserve in Canada found that men and women who did not seek the support of elders during times of need were more likely to report poorer perceived mental health, experience mood and anxiety disorders, and have suicidal thoughts or attempt suicide [24]. Other studies have found that acts of intergenerational solidarity such as family caregiving for older adults or grandmothers caring for grandchildren may have positive physical and emotional health impacts on those providing care, including lower depressive symptoms, better mental health, better quality of life, and life satisfaction [25,26]. 

In this study, we argue that negative intergenerational perceptions with regard to climate change may give rise to ageism toward older generations, which may impact their mental, physical, and social health. The same may also hold true for younger generations who depend on older generations for climate action and have been vocal about their fears regarding their health and survival in the future. Positive intergenerational perceptions, on the other hand, may increase empathy for the concerns raised by different age groups and foster cooperation among all age groups to act for the common benefit of all.

## 2. The Present Study

As opinions surrounding responsibility for the present and future states of the planet continue to be debated, the present study seeks to (1) situate the climate movement in the context of intergenerational relations to consider both the young and the old as potential victims of tension and conflict in climate change discourse, and (2) highlight the need for and scope of intergenerational cooperation and solidarity in the combined global effort to address climate change.

The term “generation” has been conceptualized variously over time but is broadly understood as “a birth cohort or group of birth cohorts who are recognised as having some common attributes and experiences by virtue of the time into which they were born” ([27], p. 99). In the context of this study, the focus is on the different generations that are currently being and/or will be affected by climate change impacts in the future. The “younger generation”, therefore, comprises those who are expected to spend more than half of their lives adapting to climate change and whose futures are likely to be shaped significantly by irreversible environmental degradation. “Older generations” comprise those above age sixty, many of whom hold immense political power with respect to climate change-related decision making. The “middle generations” comprise a broad and slightly fluid category. They are included in the term “adults”, which includes everybody over the age of 18. They have voting rights and, therefore, at least some influence in climate change-related decision making.

As we explore intergenerational sentiments and perceptions with regard to climate change, it is important to consider that generational conflict or inculpation of other generations, especially younger generations, is a fairly common social phenomenon that has been in practice for centuries. In current times, younger generations are accused of being lazy, entitled, self-obsessed, disrespectful to elders, less-resilient, less-hardworking, and weak “snowflakes” compared to older generations [28]. Conversely, older generations are commonly held responsible for the creation of nuclear weapons, economic inequalities, war, the housing crisis, the cost-of-living crisis, youth unemployment, problems regarding the welfare state, the deteriorating mental health among younger populations, and the climate crisis [29]. The difference between the two types of accusations, even though they may be unfair to large sections of the population, is a matter of perception. For example, even if younger people live up to the labels that are associated with them, they would only be harming themselves. On the other hand, the accusations leveled at older generations indicate (deliberate) large-scale, even global, damage and insecurity, especially in light of the fact that it is the older generations who wield the power to arrest and reverse a great deal of the damage. This study, therefore, reflects this generational tug-of-war regarding perceptions about climate responsibility. 

## 3. Materials and Methods

### 3.1. Data Collection and Extraction

The data collection strategy was driven by the specific intention of highlighting intergenerational relations in the context of climate change. Our goal was not to conduct an exhaustive search of all climate activists and their publicly available statements. Rather, we decided to incorporate a small representation of the different opinions held by climate activists from around the world for the explicit purpose of highlighting the variety of perspectives. In this study, the term “activist” has been used to encompass any influential individual, regardless of age, gender, or profession, who advocates for climate action. The rationale behind selecting prominent individuals lay in their popularity, recognition, influence, visibility, geographic spread, and wide outreach via social media. 

Data were collected in two stages using a purposive sampling method. In the first stage, the researchers used the Google search engine to generate lists of prominent climate activists. This was undertaken using the following search terms:

1. Influential climate activists around the world;
2. Leading climate activists;
3. Popular environmental activists;
4. Prominent climate activists;
5. List of climate warriors.

These search terms generated results such as “19 youth climate activists you should be following on social media” and “Meet 15 women leading the fight against climate change”. At this stage, the researchers retained about 40 names (after removing duplicates) that appeared on such lists. In the second stage of data collection, the researchers identified activists from climate change-related news articles. At this stage, the researchers retained the names of those who are influential and widely known (for example, New Zealand’s Prime Minister Jacinda Ardern was included based on her statement in December 2020 regarding her government’s commitment to becoming carbon-neutral by 2025 in the interest of future generations). Approximately 10 individuals were identified at this stage. Both stages of the search were undertaken from October–December 2020.

While remaining cognizant of the goal of the study, i.e., to present an overview of intergenerational perceptions in the context of climate change, the researchers shortlisted 50 individuals. We included activists of all ages, but specifically youth, as the current climate movement is largely perceived as being led by younger age groups. At the same time, we included others who, in their capacities as world leaders, celebrities, citizens, and professionals, advocate for climate action due to intergenerational concerns. We focused especially on well-known names due to their influence on large audiences and the potential for intergenerational messages to spread far and wide among their social media followers. 

Between 10 and 20 sources of information were examined for each activist. News articles, interview transcripts, magazine articles, official websites, Twitter feeds, videos, and relevant websites were scanned to gain an overview of each individual’s stance on climate activism, their work in their area of interest/expertise, and their views on intergenerational issues in the context of climate change. Only those statements that specifically referred to intergenerational relations were included; statements addressing the need for climate action in general were not included. 

The data were collated in a tabular form to create profiles for each activist. A list of selected individuals is available in Supplementary Table S1. Data collection included the following components: (a) name; (b) age; (c) sex; (d) activist type; (e) country; (f) World Bank country classification; (g) profession; (h) scope of work; (i) topic(s) of interest/main issue(s) addressed; (j) notable accomplishments; (k) personal/other initiative; (l) intergenerational focus; (m) views regarding “who/what is responsible for climate change?”; (n) number of followers on Twitter, Instagram, Facebook, and YouTube (personal pages/channels); (o) books/blogs; (p) unique contributions; and (q) other relevant remarks. Of these, the component “views regarding who/what is responsible for climate change?” was used for data analysis. 

### 3.2. Data Analysis

A summative content analysis method was used to analyze the data [30]. It was applied to the components that focused on intergenerational relations with regard to who or what is responsible for climate change and who is affected by climate change. The summative content analysis approach is particularly suited to the understanding and interpretation of textual content from the use of specific words. Since this study focused on intergenerational relations, this method was used to code messages using the following terms: “adults”, “older”, “old”, “young”, “youth”, “younger”, “children”, “kids”, “leaders”, “politicians”, “corporations”, “interest groups”, and “future”. This provided an overview of what/who is commonly considered responsible for the current climate crisis (for example, large corporations and politicians with vested interests in polluting industries) and who is most affected by climate change. The researchers then categorized the statements into two broad categories: (1) intergenerational tension or conflict and (2) intergenerational cooperation or solidarity. Quotes that accused other generations of perpetuating or ignoring climate change were placed in the first category and quotes that offered positive intergenerational sentiments were placed in the second category. These two broad categories were subsequently refined to provide a more nuanced understanding of intergenerational relations in the context of climate change. These are presented in the Findings section.

## 4. Findings

Relying on a scoping review, which proposed two arms for intergenerational relations [7], we aimed for a more nuanced understanding of these arms starting from two divergent types of public messaging: messages depicting intergenerational tension and inclusive messages of intergenerational solidarity. Messages of intergenerational tension harbor the potential to increase hostility between different generations, whereas messages of intergenerational solidarity may lead to increased empathy and understanding of common struggles regardless of age and generation. An exploration of these two types of messaging allows for a holistic understanding of how the climate movement is viewed by different stakeholders in the context of intergenerational relations. 

The Present Sample:

The activists identified for this study included youth activists, scientists, politicians, lawyers, actors, architects, diplomats, entrepreneurs, TV presenters, peace ambassadors, environmental/racial/indigenous people’s justice campaigners, celebrities, and several other professionals and members of civil society. Overall, the activists comprised 50 individuals with an average age of 30.97 years (based on the available ages of 45 individuals); the age range was 12–94 years. Twenty-eight people were below age 30, eleven were between ages 30 and 60, and six were over age 60. The majority of individuals were female (39; 78%), while 11 (22%) were male.

### 4.1. Messages of Intergenerational Tension

Messages of intergenerational tension comprise statements that explicitly blame adults and older generations for the current state of the climate. Such messages have their genesis in multiple factors, including (1) past actions that have exacerbated climate change to its present state of crisis, (2) current inaction that will almost certainly endanger the futures of current younger generations, (3) the suppression of youth voices concerning climate matters, and (4) the limited ability of younger people to effect tangible changes in the global order. Youth activists, many of whom are below 18 years of age, are acutely aware of their lack of voting rights. Consequently, the furthest possible extent of their influence involves pressuring governments and global bodies into taking action and holding them accountable for their inaction/inadequate action. Often, they are ridiculed for their demands and dismissed as “misled” or “idealistic” by sections of the media and powerful people. 

In this study, we did not find evidence of negative sentiments towards youth activists/younger generations, possibly because many of the selected individuals were youth activists themselves. Therefore, as illustrated below, this section refers specifically to instances of younger generations blaming (older) adults for the climate crisis. The quotes emphasize tensions between the present and the future and between different generations; portray children as de facto leaders in a situation wherein they are clearly the victims; and show how children are forced to take drastic measures, such as regularly skipping school, to highlight that which is obvious to the majority of the global population.

You say you love your children above all else, and yet you are stealing their future in front of their very eyes. Until you start focusing on what needs to be done rather than what is politically possible, there is no hope. We cannot solve a crisis without treating it as a crisis

(Greta Thunberg, 17, Sweden)

Thousands of children around the world should not be having to miss classes because of our leaders’ inability to treat the climate crisis as a crisis.

The very point about missing school is that it makes people realize it’s important and we’re willing to sacrifice an hour of education a week

(Holly Gillibrand, 14, Scotland)

When the United Nations announced it was giving Fridays for Future one of its Champions of the Earth awards, we said we would hold the award, to give back to our leaders when they actually took action. Because in our view, we’re kind of the facilitators to grow the political will. But they’re the ones that are really going to have to make large-scale change

(Kallan Benson, 16, United States)

Youth climate activists understand the need for political will and societal sacrifice in order to bring about large-scale changes. Since they are ineligible to vote, they try to make their voices heard in other ways. For example, 12-year-old Lilly Platt from the Netherlands who founded Lilly’s Plastic Pickup urged her grandfather to vote for her in the latest election because she “wasn’t of age to vote for someone that does great things for the planet”. She subsequently made a video to share with the world because she believes that “adults should let children speak”. Youth climate activists generally stop short of explicitly laying the blame for climate inaction on any one generation. Instead, acting as mobilizers of adults and older generations, they want voters, politicians, and leaders, irrespective of age, to make the right decisions. However, as illustrated by the quote below, since most leaders and decision-makers in high offices and politicians with influence happen to be older adults, the climate movement may come to be construed as ageist toward older adults.

Older people bear much more of the responsibility than the younger generations. For young people, this means turning to those who have responsibility and make the decisions. For the older generation, this means understanding that they are part of the solution. It’s sobering to see the amount of effort we need to maintain an already-negotiated intergovernmental agreement so that we don’t miss the Paris climate targets

(Luisa Neubauer, 24, Germany)

However, not all young activists are equally circumspect. For example, in 2019, 19-year-old American singer Billie Eilish made the following remarks: “Hopefully the adults and the old people start listening to us [about climate change] so that we don’t all die. Old people are gonna die and don’t really care if we die, but we don’t wanna die yet.” Similarly, Greta Thunberg and fellow youth activists alleged that, “Young people are being let down by older generations and those in power” at Davos in 2020. Although Eilish isolates “old people” for their supposed indifference toward young people’s lives, youth activists, in general, seem to use the term “older generations/people” to encompass all adults who have the power to vote and/or effect large-scale change. Consequently, they unanimously hold politicians, world leaders, large corporations, and interest groups responsible for the state of the planet. They also often express disillusionment with respect to the lack of political will displayed by various governments. Most youth activists realize that they will inherit the burden of mitigating climate change; many are looking forward to the time when they will become voters and leaders:

With the youth movement growing so quickly throughout the world, it’s exciting to see our power and influence also grow as the attention is more and more focused on us. This gives me hope, and I look forward to seeing where it will lead in future years as we become old enough to vote and hold jobs in which we have power over fundamental decisions

(Isabel “Scout” Pronto Breslin, 16, United States)

Although such optimism and resoluteness on the part of younger people bodes well for the future, the window is rapidly closing on the remaining time in which to limit global warming to 1.5 degrees. For now, however, as younger generations hold limited political power and older generations exercise limited political will, the climate movement remains embroiled in a political and economic tug of war, and the agreed upon climate goals continue to remain unfulfilled.

### 4.2. Messages of Intergenerational Solidarity

The messages of intergenerational solidarity were similar to those of intergenerational tension in that they recognized the same reality that past (in)action has generated consequences for the present and that present (in)action will determine the future. Additionally, the messages of intergenerational solidarity focused on younger generations as victims and older generations as perpetrators as well as change-makers. The primary difference between messages of intergenerational tension and solidarity was that in the former, the younger generation felt the need to indicate to older generations their own culpability in the climate crisis, whereas in the latter, the older generations accepted responsibility for the present and the future by bearing witness to the changes that the planet has undergone under their watch. Moreover, by accepting responsibility, older generations lent legitimacy to young people’s narratives and accorded due respect to their concerns.

The messages of intergenerational solidarity, therefore, were generally imbued with a spirit of empathy and collaboration. These messages were primarily offered by members of older age groups in relation to the futures of their children, grandchildren, and younger generations in general. Such individuals believed that it is incumbent upon older generations to act to the full extent of their professional and personal capacities in order to leave behind a planet that will allow future generations to thrive instead of suffer the consequences of the previous generations’ (in)actions. In this way, they often transformed potentially incendiary sentiments to those of understanding and compassion.

Our responsibility is to leave our home better than we found it so that future generations, no matter where they are born, or to whom, can thrive.

I saw a species literally disappear in my lifetime. We don’t become mothers to hand over something that is diminishing to our children.

Greta [Thunberg] in her usual style delivered a scathing speech about the irresponsibility with which we have been non-acting and was very, very clear about if we don’t do our job she and her generation will not forgive us, so just swallow that one for a moment

(Christina Figueres, 64)

Ms. Figueres, a Costa Rican diplomat widely credited for negotiating the Paris Agreement in 2015, belongs to the generation that is accused by youth activists of endangering the planet by misusing and/or underutilizing their authority and influence in government and corporate offices, as well as leading unsustainable lifestyles that are harmful for the health of the planet. Ms. Figueres, however, recognizes the valid concerns expressed by the younger generation and, instead of dismissing them for being young, uses her position to mobilize governments to take concrete action. 

Another example of intergenerational solidarity was offered in 2019 by António Guterres, the Secretary-General of the United Nations, who acknowledged young people’s rightful anger at older generations’ lack of response to the climate crisis. Thus, he turned a potentially inflammatory sentiment into one of intergenerational solidarity and understanding. When powerful people lend legitimacy to younger people’s fears and concerns about their survival, the message resonates with the larger community and underlines the urgency of the situation. It also harbors the potential to neutralize intergenerational tension and channel resources towards constructive, outcome-oriented goals.

These schoolchildren have grasped something that seems to elude many of their elders: we are in a race for our lives, and we are losing. The window of opportunity is closing—we no longer have the luxury of time, and climate delay is almost as dangerous as climate denial. My generation has failed to respond properly to the dramatic challenge of climate change. This is deeply felt by young people. No wonder they are angry.

Similarly, governments around the world have taken cognizance of the climate movement and lent support to the concerns of the younger generations by adopting climate-friendly legislations. For example, in December 2020, New Zealand joined 32 other countries to declare a climate emergency and committed to becoming a carbon-neutral government by 2025 so that younger generations have “a reason for hope”:

This declaration is an acknowledgement of the next generation. An acknowledgement of the burden that they will carry if we do not get this right and do not take action now. It is up to us to make sure we demonstrate a plan for action, and a reason for hope

(Jacinda Ardern, 40, New Zealand)

Acts of intergenerational solidarity were also displayed by younger generations. Although they are concerned about their own futures, they understand the need to set precedent by doing their share to protect members of older generations. Jerome Foster II, an 18-year-old American climate activist, believes that all generations must support and protect one another by making sacrifices that benefit everybody concerned, just as the current younger generation has effected by sacrificing their education to keep older generations safe during the COVID-19 pandemic:

With COVID-19…we’re seeing that young people have sacrificed their education to make sure that our elders have a livable future and can live lives that are great for them. Climate change is the reverse of that. We’re asking for older generations to fight for us and to fight for our future. We ask that you sacrifice a little bit to make sure that we have decades to also live healthy and clean. That’s the duality of climate change and the climate crisis; sometimes you have to sacrifice to make sure that everyone has the quality of life that they deserve and need.

Foster, like other youth climate activists, displays maturity, an understanding for the need for intergenerational collaboration, and the willingness to sacrifice something as important as education for the betterment of all. He uses the COVID-19 pandemic as an example of large-scale intergenerational solidarity and collaboration whereby one generation sacrificed something that is essential to its wellbeing to keep another generation safe at a critical time for the world. Although this sacrifice by young students to protect members of older generations had the potential to transform into an intergenerational conflict, the youth have used the pandemic to exemplify their stance on intergenerational cooperation and to reiterate their belief in science by heeding the warnings of governments and the medical community. Thus, they set an example for other generations in terms of following the science concerning climate change, paying heed to warnings, and making necessary sacrifices so that all generations, especially younger and future generations, have a chance at survival. 

## 5. Discussion

This study was an effort to consolidate differing worldviews on intergenerational relations in the context of climate change. Specifically, we aimed to examine statements made by well-known, influential activists and people in positions of power to highlight how these might impact public perceptions about climate responsibility. We categorized the selected rhetoric into “statements of solidarity” and “statements of conflict” between generations. Our findings highlight similarities between the two types of messaging as both acknowledge and address the same underlying issue: climate change and the need for immediate action. These are signs of hope and indicate the potential for intergenerational collaboration as there is a general agreement on the facts. However, the public expression and presentation of the problem may be a cause for concern since these often explicitly blame large sections of the population for the climate crisis. 

Both activists that emphasize tension and conflict and those who stress solidarity between the generations highlight the short window of opportunity and the need to act in the present for the safety and prosperity of future generations. Moreover, both sides emphasize the responsibility of older people, and adults in general, to act, given the fact that they are the ones who hold the power to make changes. Both sides also acknowledge the limited political power of children and youth, which places them at a position in which they must depend upon (older) adults to represent their interests.

The call for climate action rests primarily upon the concepts of intergenerational equity and justice [4,5]. It calls for a moral and ethical understanding of the current responsibilities vis-à-vis a livable future for younger and unborn generations. Although it now appears to be beyond our capacity to undo the existing damage to the planet, it is considered incumbent upon current generations to at least mitigate the predicted harm in the foreseeable future [31]. For years, studies around the world have arrived at the common conclusion that (1) anthropogenic climate change exists, (2) it needs to be addressed with immediate effect, and (3) if left unattended, future generations and socially and geographically vulnerable populations will be the most severely affected by this phenomenon. However, despite repeated and dire warnings, much work remains to be performed in order to alleviate these justifiable fears and concerns, a fact that has been recognized and repeatedly highlighted by activists.

Since the climate movement gathered impassioned support in 2018 when teen activist Greta Thunberg inspired young people from around the world to demand climate action, activists, especially youth activists, have faced multiple hurdles in their efforts to raise awareness about climate change. From being dismissed for their age to being belittled for their cognitive development, youth activists have experienced extreme forms of ageism on the global stage [32]. In addition to ageism, they have also grappled with sexism, racism, and bullying by adults, including by powerful leaders and politicians [33]. The struggles of youth climate activists are reflective of the issues faced by youth activists in other spheres as well. Such issues include feeling misunderstood, exploring the meaning of youth activism and its influence and relevance, and desires and expectations pertaining to intergenerational cooperation and allyship [34]. In many ways, adults with vested interests have tried to suppress the voices of youth activists by attacking, first and foremost, their age and then by associating their youth with a lack of practical, worldly knowledge. Having been at the receiving end of such unjust stereotypes, prejudices, and discrimination, young activists have turned against older generations, especially those occupying powerful positions. In their effort to shine light upon such injustices and climate inaction, many activists have taken refuge in sentiments that may negatively impact intergenerational relations. A recent study has found that negative views about older adults may motivate people to deeply consider the impacts of climate change and increase their willingness to pay higher taxes in support of climate action [35]. Negative perceptions, however, may also lead to increased ageism towards older adults who might feel unfairly victimized and withdraw support from climate action, thereby increasing the risk to the health and wellbeing of all. We suggest here, therefore, that the same messages about climate change impact and climate responsibility can be delivered to foster solidarity and support between the generations. 

Unlike messages of intergenerational conflict that carry the potential to fan hostilities, messages of intergenerational solidarity are built on compassion, empathy, understanding, consideration, cooperation, respect, trust, and hope. Nonetheless, these messages also represent a call for action and emphasize the responsibility of older generations toward the welfare of younger generations. In this study, the two kinds of statements presented a stark contrast to one another in terms of rhetoric but not in the underlying messages. This highlights the power of inclusive discourse to unite people across generations to act out of a sense of urgency. 

Recently, the COVID-19 pandemic served as a timely reminder of the degree of sacrifice required when the world has exhausted all its options. In a similar vein, if we do not act on climate change now, the sacrifices that will be required later will be much more intense and prolonged. As shown in this article, many (older) adults have taken it upon themselves to support the climate fight on behalf of younger generations, even though they themselves will not reap the direct benefits of their actions. Although much of this solidarity stems from their concerns for their children and grandchildren, it may also be a conscious effort on their part to deflect the blame and accusation that they receive from younger generations. Whatever the reason, language accusing (older) adults of climate degradation may not only be perceived as unfair to individuals who are genuinely invested in the betterment of the planet, but it may also alienate well-meaning allies—with a considerable share of voting power—in the climate fight. Hence, such language is counter-productive, especially given the similarities in the underlying content found across the two poles of rhetoric that emphasizes conflict versus solidarity. 

It is also important to remember that whatever little climate action has been undertaken so far, in terms of policy decisions, the gradual global shift toward clean energy, extensive research on climate friendly products and practices, large-scale awareness about climate change, and individual efforts to help the planet have not been achieved without the work and support of (older) adults. Although these efforts may still be insufficient, they are on-going, with intentions of being scaled up. Therefore, it is only fair to acknowledge the contributions of older generations toward climate action and reiterate the fact that everybody needs to act in order to turn the climate crisis around because everybody stands to lose from this unprecedented emergency. 

However, it is important to recognize that positive messages on social media are not enough to alter perceptions or mobilize generations to work together. Other, more concrete steps must be taken to harness the political will, passion, and interests of all generations. One way to achieve this may be to invest in intergenerational contact/interventions for climate action. Intergenerational contact comprises many forms. Formal contact may include structured volunteering programs, recreational programs, daycare programs, and educational programs. Informal settings may include spending time with family members, relatives, or friends of different age groups. At the community level, Intergenerational Contact Zones in parks, community centers, community gardens, reading rooms, clubhouses, and other shared community spaces are gradually gaining popularity [36] Regardless of the type of contact, the quantity (long-term, sustained contact) and quality (opportunities for developing close interpersonal relationships) of contact are important components of successful outcomes [2], which may include reduced prejudice and greater respect toward older generations [37,38,39], positive attitude of older adults toward younger generations [40,41,42], and enhanced interest among different generations with respect to working together on varied environmental projects [7]. Sustained effort and investment in intergenerational contact and collaboration toward climate action may serve the dual purpose of increasing respect for the abilities and interests of all age groups as well as contributing to the larger fight against climate change. These actions may also help to address ageism toward both young and old, which, in turn, may lead to positive physical, mental, and social health outcomes. Intergenerational contact initiatives, therefore, must form an integral part of climate adaptation and mitigation strategies around the world. 

In this study, we intended to provide an overview of the different types of messaging in climate discourse with regard to intergenerational relations. Since it was not conceptualized as a comprehensive study on contemporary climate discourse, it has several limitations. First, we selected only a handful of activists from amongst thousands around the world. We relied on lists generated by Google to shortlist those who are famous and have greater influence than others since such individuals can shape the thoughts of large numbers of people. Although we did not include climate leaders who do not have a large social media following, we recognize that many such people work at grassroots levels and that their contributions are equally important. Moreover, most of the activists that we selected (which appeared on the Googled lists) were youth activists. Therefore, most of the data we examined presented the subject of discussion from the perspectives of young people. Consequently, older generations and their perspectives on the matter are underrepresented in this study. The second limitation indicates the lack of regional diversity. Most activists on our list belong to developed countries, whereas climate change is expected to have more severe consequences for developing nations. Therefore, it is imperative to consider the challenges being faced by people in low- and middle-income countries. The third limitation is related to the methodology adopted for the study. Since we did not intend to carry out an exhaustive search and analysis of contemporary climate discourse, we approached the data as readers or consumers of the most widely circulated social media messages/news reports by the most influential individuals. This allowed us to gain an overview of the most visible sentiments regarding intergenerational relations and climate responsibility. A future study that is intended to explore contemporary climate discourse more thoroughly would benefit from a systematic approach to data collection, regardless of the popularity of an individual or their social media outreach, and a detailed analysis accounting for the experiences of activists at local, national, and international levels and including more activists from developing nations. 

## 6. Conclusions

This study, on the one hand, demonstrates how the climate movement has polarized people across age groups on grounds of political decision making and global leadership. On the other hand, it aims to elucidate the climate fight as a necessarily united effort that will allow all generations to live up to their potential. This can only happen when all parties’ efforts are invited, shared, implemented, acknowledged, and appreciated. Clearly, climate change’s effects and mitigation efforts have important temporal aspects that cannot be denied. Nonetheless, our findings highlight the potential to address these temporal aspects by bringing generations together toward a common goal. It is also important to give credit where it is due, namely, to younger people for leading the climate fight and to older generations for mobilizing resources to act on the latest climate science. Most importantly, it is imperative that people of all ages understand the need for cooperation and collaboration at this critical time. Intergenerational discourse that alienates collaborators, sympathizers, and well-wishers must be replaced with messages of inclusivity highlighting the potential for “all-hands-on-deck” opportunities. All parties would benefit from careful consideration of the language that they use to inspire everyone, regardless of age, to “unite behind the science” for the benefit of all.

## Data Availability

The data included in the article are available to the public on the internet. Sources of data are listed in Table S1.

## Supplementary Materials

The following supporting information can be downloaded at: https://www.mdpi.com/article/10.3390/ijerph20010233/s1, Table S1: List of activists.
