# Supplementary material for: Intergenerational Relations in the Climate Movement: Bridging the Gap toward a Common Goal
Source: Int J Environ Res Public Health. 2022 Dec 23;20(1):233. doi: 10.3390/ijerph20010233 (PMC9819550; doi:10.3390/ijerph20010233)
Supplement: Supplementary file 1 [file ijerph-20-00233-s001.zip › ijerph-1957434-supplementary.pdf]

Supplementary Table S1

Supplementary Table S1. List of activists.

Sr. No.	Name	Age	Sex	Country
1	Christiana Figueres	64	F	Costa Rica
2	Rhiana Gunn-Wright	32	F	United States
3	Hilda Heini	69	F	Marshall Islands
4	Hindou Oumarou Ibrahim	36	F	Chad
5	Tessa Khan		F	Australia
6	Rachel Kyte		F	United States
7	Kate Marvel		F	United States
8	Sunita Narain	59	F	India
9	Ellen Page	33	F	Canada
10	Anne Simpson		F	United States
11	Greta Thunberg	17	F	Sweden
12	Kotchakorn Voraakhom	39	F	Thailand
13	Miranda Wang	26	F	United States
14	Katharine Wilkinson	37	F	United States
15	Wu Changhua		F	China
16	Bill Nye	46	M	United States
17	Al Gore	72	M	United States
18	Marinel Sumook Ubaldo	23	F	Phillippines
19	Xiye Bastida	18	F	Mexico
20	Kallan Benson	16	F	United States
21	Vic Barrett	21	F	United States
22	John Paul Jose	23	M	India
23	Luisa Neubauer	24	F	Germany
24	Isra Hirsi	17	F	United States
25	Holly Gillibrand	14	F	Scotland
26	David Wicker	15	M	Italy
27	Xiuhtezcatl Martinez	20	M	United States
28	Lilly Platt	12	F	The Netherlands
29	Saoi O’Connor	17	F	Ireland
30	Jamie Margolin	18	F	United States
31	Leah Namugerwa	15	F	Uganda
32	Anuna De Wever	19	F	Belgium
33	Jerome Foster II	18	M	United States
34	Eyal Weintraub	19	M	Argentina
35	Alexandria Villaseñor	15	F	United States
36	Haven Coleman	13	F	United States
37	Milou Albrecht	15	F	Australia
38	Jayden Foytlin	17	F	United States
39	Isabel (Scout) Pronto Breslin	16	F	United States
40	Noga Levy-Rapoport	18	F	United Kingdom

41	Severn Cullis-Suzuki	40	F	Canada
42	Delaney Reynolds	18	F	United States
43	Jacinda Ardern	40	F	New Zealand
44	Sir David Attenborough	94	M	United Kingdom
45	Alexandia Ocasio-Cortez	31	F	United States
46	Bernie Sanders	79	M	United States
47	Leonardo Di Caprio	46	M	United States
48	Billie Eilish	19	F	United States
49	Disha Ravi	22	F	India
50	António Guterres	72	M	Portuguese
